# Analysis of HBV Genomes Integrated into the Genomes of Human Hepatoma PLC/PRF/5 Cells by HBV Sequence Capture-Based Next-Generation Sequencing

**DOI:** 10.3390/genes11060661

**Published:** 2020-06-18

**Authors:** Tomotaka Ishii, Akinori Tamura, Toshikatsu Shibata, Kazumichi Kuroda, Tatsuo Kanda, Masaya Sugiyama, Masashi Mizokami, Mitsuhiko Moriyama

**Affiliations:** 1 Division of Gastroenterology and Hepatology, Department of Medicine, Nihon University School of Medicine, 30-1 Oyaguchi-kamicho, Itabashi-ku, Tokyo 173-8610, Japan; ishii.tomotaka@nihon-u.ac.jp (T.I.); tamuraakinori0306@yahoo.co.jp (A.T.); toshimei@gmail.com (T.S.); kuroda.kazumichi@nihon-u.ac.jp (K.K.); moriyama.mitsuhiko@nihon-u.ac.jp (M.M.); 2 Genome Medical Science Project, National Center for Global Health and Medicine, Ichikawa 272-8516, Japan; msugiyama@hosp.ncgm.go.jp (M.S.); mizokami0810@gmail.com (M.M.)

**Keywords:** chromosome, HBV, HCC, hepatocarcinogenesis, Huh7, integration, next-generation sequencing, PLC/PRF/5, transfection

## Abstract

Hepatitis B virus (HBV) infection is a leading cause of hepatocellular carcinoma (HCC) worldwide. The integration of HBV genomic DNA into the host genome occurs randomly, early after infection, and is associated with hepatocarcinogenesis in HBV-infected patients. Therefore, it is important to analyze HBV genome integration. We analyzed HBV genome integration in human hepatoma PLC/PRF/5 cells by HBV sequence capture-based next-generation sequencing (NGS) methods. We confirmed the results by using Sanger sequencing methods. We observed that HBV genotype A is integrated into the genome of PLC/PRF/5 cells. HBV sequence capture-based NGS is useful for the analysis of HBV genome integrants and their locations in the human genome. Among the HBV genome integrants, we performed functional analysis and demonstrated the automatic expression of some HBV proteins encoded by HBV integrants from chromosomes 3 and 11 in Huh7 cells transfected with these DNA sequences. HBV sequence capture-based NGS may be a useful tool for the assessment of HBV genome integration into the human genome in clinical samples and suggests new strategies for hepatocarcinogenesis in HBV infection.

## 1. Introduction

Hepatitis B virus (HBV) infection occasionally induces hepatocellular carcinoma (HCC) through direct and indirect mechanisms and is an important cause of morbidity and mortality worldwide. HBV is a partially double-stranded DNA hepatotropic virus of ~3.2 kb in length. The HBV DNA sequence consists of four open reading frames encoding the surface (HBsAg), core (HBcAg), polymerase, and X (HBx) proteins [1,2].

Soon after HBV infection, HBV DNA is converted into a covalently closed circular DNA molecule (HBV cccDNA) in the nucleus of HBV-infected cells as a stable episomal template [3]. HBV cccDNA is responsible for the chronic persistent HBV infection of hepatocytes. On the other hand, the integration of HBV DNA into the host genome occurs randomly, early after infection, and seems to continue [4,5,6,7,8]. Although the frequent observation of somatic integration of HBV DNA suggests a possible benefit for HBV replication, the mechanism of integration, its functions, and the clinical impact on hepatocarcinogenesis remain largely unknown [9,10].

Next-generation sequencing (NGS) has been applied in various fields of virology and cancer research [6,7]. However, the low abundance of viral integration occasionally prevents viral identification. Specific target-sequence capture-based NGS has been developed as a method for detecting low levels of virus particles [11]. After fragments with HBV sequence were enriched by a set of HBV probes, high-throughput sequencing could detect the location of HBV integration breakpoints in the HCC genome [12]. Capture sequencing methods have higher sensitivity and efficiently detect sequences with low costs, compared to conventional methods [12,13].

In this report, we analyze HBV genome integration in human hepatoma PLC/PRF/5 cells [14] by more sensitive HBV sequence capture-based NGS methods. We constructed an HBV genome sequence and determined the HBV genotype (GT) in human hepatoma PLC/PRF/5 cells. We also focus on the topical subject of HBV DNA integration, which is linked HCC progression. We reveal HBV DNA integrants and their locations in the human genome. HBV sequence capture-based NGS is a useful and powerful tool for the assessment of HBV genome integration.

## 2. Materials and Methods

### 2.1. Cell Culture

Human hepatoma PLC/PRF/5 and Huh7 cells were purchased from the Japanese Collection of Research Bioresources (JCRB) Cell Bank (Osaka, Japan) [14]. The cells were maintained in Dulbecco’s modified Eagle’s medium (DMEM, Sigma-Aldrich, St. Louis, MO, USA) supplemented with 10% fetal bovine serum and 100 U/mL penicillin/100 μg/mL streptomycin at 37 °C in a 5% CO_2_ atmosphere. We used PLC/PRF/5 cells within 5 passages in the present study.

### 2.2. Cellular DNA Extraction and DNA Fragmentation

Total DNA was isolated using a QIAamp DNA Mini Kit (Qiagen, Tokyo, Japan) according to the manufacturer’s instructions. A total of 10 μg DNA was fragmented using an E-200 ultrasonicator (Covaris, Unit H. Woburn, MA, USA) [15], and the size of the resulting DNA fragments was ~300 bp, which was confirmed by using an Agilent Bioanalyzer 2100 (Agilent Technologies, San Jose, CA, USA).

### 2.3. Library Preparation and HBV Sequence Capture-Based Next-Generation Sequencing (NGS)

After the generation of blunt-ended fragments, a sequence adapter was added with a TruSeq Nano DNA Sample Prep Kit (Illumina, San Diego, CA, USA). Target-capture sequencing on the Roche SeqCap platform was performed across HBV full genomes: GT-A (AP007263), GT-B (AB287327), and GT-C (AB368296). The DNA libraries from PLC/PRF/5 cells were captured using probes generated from these 3 HBV sequences by using SeqCap EZ Libraries (Roche NimbleGen, Tokyo, Japan).

HBV DNA-specific fragments were selectively collected with Invitrogen Dynabeads (Invitrogen, Carlsbad, CA, USA) and used as genome libraries. NGS of these libraries was performed by using a HiSeq 2000 (Illumina) system with a HiSeq PE Cluster Kit cBot (Invitrogen). Sequence data were processed using a standard pipeline in CLC Genomics Workbench (Qiagen). We focused on the accumulation of HBV-integrated sequences/fused/chimeric sequences and analyzed the sequences of the HBV and human genomes in each read by HBV sequence capture-based NGS. The integrated sequences of the HBV and human genomes with sequence reads >100 were analyzed.

HBV short genome sequences isolated at NGS were mapped to the reference sequence of HBV full genome GT-C (AB014378), resulting in the full-length HBV sequence (PLC-HBV) derived from PLC/PRF/5 cells being obtained.

All sequence reads have been submitted to the DNA Data Bank of Japan (DDBJ; temporary submission ID: SSUB015333).

### 2.4. Confirmation of HBV Genome Integration by Sanger Sequencing Methods

PCR primers were designed at the following locations in the human genome (hg19): 131170441–131170444 and 131172081–131172105, 64808026–64808415 and 64808438–64808453, and 80063555–80063577 and 80065509–80065534 for HBV integrants in chromosomes 3, 11, and 17, respectively. PCR primers were also designed at the following locations, the human genome (hg19): 1296892–1296914 on chromosome 5 and 33662450–33662472 for translocation of chromosome (5; 13). A total of 100 ng DNA was amplified by PCR with KOD FX Neo (KFX-201, Toyobo, Osaka, Japan), according to the manufacturer’s instructions.

The PCR products were cloned into the pCR-Blunt II-TOPO vector (Invitrogen). Sanger sequencing was performed with M13 forward and reverse primers using the BigDye Terminator v3.1 Cycle Sequencing Kit (Thermo Fisher Scientific, Tokyo, Japan) and an ABI 3730xl DNA Genetic Analyzer (Thermo Fisher Scientific), according to the manufacturer’s instructions.

### 2.5. Phylogenetic Analysis

GENETYX version 10 (GENETEX Corp., Shibuya, Tokyo, Japan) was used to analyze the nucleotide sequences and perform phylogenetic tree analysis by neighbor-joining (NJ) methods. The statistical reliability of the phylogenetic trees was assessed using the bootstrap method on 10,000 times and the Kimura two-parameter model [16]. The accession numbers of sequences of various HBV GTs are indicated [17,18,19,20,21].

### 2.6. Immunofluorescence Study

HBV integrants from chromosomes 3 and 11 were cloned into the pCR-Blunt II-TOPO vector. Plasmids were transfected into Huh7 cells using Lipofectamine 2000 (Thermo Fisher Scientific). After 24 h of transfection, the cells were fixed with 4% paraformaldehyde (Gibco, Palo Alto, CA, USA) and incubated with an HBsAg-specific mouse monoclonal antibody (M3506, Dako, Carpinteria, CA, USA) at a dilution of 1:50, or HBV polymerase-specific mouse monoclonal antibody (2C8, sc-81590, Santa Cruz Biotechnology, Dallas, TX, USA) at a dilution of 1:50 for 1 h. The cells were washed and incubated with anti-mouse Ig conjugated with an Alexa 594 secondary antibody (Invitrogen) at a dilution of 1:500 for 1 h at room temperature. Finally, the cells were washed and mounted for fluorescence microscopy (BIOREVIO BZ-9000, Keyence, Osaka, Japan). PLC/PRF/5 cells were used as control.

## 3. Results

### 3.1. HBV DNA Sequence Derived from PLC/PRF/5 Cells

Using HBV sequence capture-based NGS technology, we analyzed PLC/PRF/5 cells to reveal the HBV signature. HBV genome sequences were found in PLC/PRF/5 cells (1,784,734 reads out of 6,165,629 reads, including the mitochondrial genome; see Table 1). We mapped these sequences onto a previously reported HBV sequence to obtain the full-length HBV sequence (PLC-HBV) derived from PLC/PRF/5 cells. PLC-HBV has been deposited in the DNA Data Bank of Japan (https://www.ddbj.nig.ac.jp/index-e.html; accessed on 28 March 2020 under accession number LC533934).

**Figure 1:**
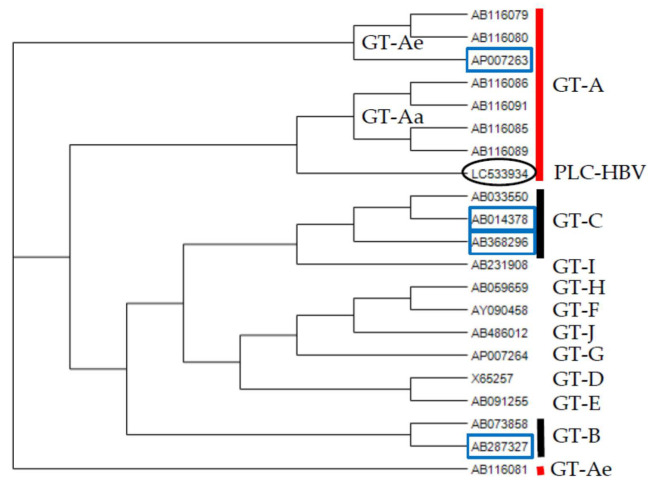
Phylogenetic tree for the hepatitis B virus (HBV) full-length genome obtained in the present study as determined by neighbor-joining (NJ) methods. **Black circle**, PLC-HBV (LC533934); **blue square**, reference sequences in the present study. GT-Ae, the original European genotype A; GT-Aa, the new African/Asian genotype A. The accession numbers of sequences of various HBV genotypes (GTs) are indicated [21,22,23,27,28].

Phylogenetic tree analysis by the neighbor-joining (NJ) method demonstrated that PLC-HBV belongs to HBV GT-A (Figure 1), in agreement with the production of subtype adw of HBsAg by PLC/PRF/5 cells [14,17] and the finding that the HBV genome sequences recovered from PLC/PRF/5 cells belong to HBV GT-A [18]. PLC-HBV shows nucleotide homology of 94% (3054/3221), 90% (2919/3220), and 91% (2932/3220) to the HBV sequences used for the generation of HBV capture probes in the present study (GT-A, HB-JI444AF (AP007263); GT-B, JPN Bj A53 (AB287327); GT-C C2, HBV-CH48-201w (AB368296), respectively) [22,23].

PLC/PRF/5 cells do not harbor an intracellular free HBV genome or infectious HBV virions in the conditioned medium [19,24,25,26]. Although there is one report showing that PLC/PRF/5 cells harbor full-length HBV genomes [20], the PLC-HBV genome is constructed from multiple partial HBV genome sequences in PLC/PRF/5 cells. Here, we demonstrate that HBV GT-A is integrated into the genome of PLC/PRF/5 cells.

### 3.2. Analysis of HBV Genome Integrants and Their Locations in the Human Genome

We focused on the accumulation of HBV-integrated sequences/fused/chimeric sequences and analyzed the sequences of the HBV and human genomes in each read by HBV sequence capture-based NGS. HBV-integrated sequences/fused/chimeric sequences consist of one derived from the host and the other from HBV. The results of the analysis for the integrated sequences of the HBV and human genomes with sequence reads >100 are shown in Table 2.

**Figure 2:**
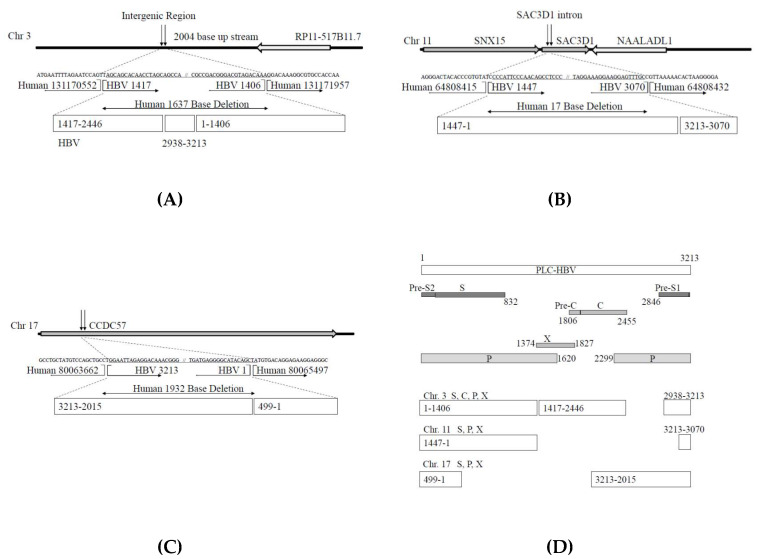
Results for integrated sequences of hepatitis B virus (HBV) and human genome chromosomes (Chrs) 3, 11, and 17 determined by Sanger sequencing. (**A**) Chr 3, (**B**) Chr 11, (**C**) Chr 17, deletion of human genome sequence including part of an exon, (**D**) summary of HBV integrants in PLC/PRF/5 cells confirmed by Sanger methods. SNX15, sorting nexin 15; SAC3D1, SAC3 domain-containing 1; NAALADL1, N-acetylated alpha-linked acidic dipeptidase such as 1; CCDC57, coiled-coil domain-containing 57; S, surface antigen; C, core; P, polymerase; X, HBx.

Figure 2 shows the results regarding the integrated sequences of HBV and human genome chromosomes 3, 11, and 17, confirmed by Sanger sequencing methods. These results were different to some extent from those of NGS. Figure 2A presents the results for integrated HBV and human chromosome 3 sequences. Human genome chromosome 3 exhibits a 1637-bp deletion with the insertion of a partial 2623-bp HBV DNA sequence. The HBV regions included in this integrant are shown in Figure 2D.

Figure 2B shows the results regarding integrated HBV and human chromosome 11 sequences. Human genome chromosome 11 exhibits a 17-bp deletion with the insertion of a partial 1591-bp HBV DNA sequence. The HBV regions included in this integrant are shown in Figure 2D. The insertion point of human chromosome 11 is the intron of the SAC3 domain-containing 1 (SAC3D1) region. The SAC3D1 mRNA is significantly associated with the overall survival of patients with HCC (B Cox value, +0.540; HR (95% CI), 1.717 (0.179–3.0 0); *p* < 0.0001). Higher expression of SAC3D1 mRNA in HCC is considered a high-risk factor associated with short survival [29]. SAC3D1 is associated with centrosome abnormalities, and SAC3D1 could be a prognostic marker for HCC recurrence after surgical treatment [30].

Figure 2C provides the results for integrated HBV and human chromosome 17 sequences. Human genome chromosome 17 harbors a 1932-bp deletion with the insertion of a partial 1699-bp HBV DNA sequence. The HBV regions included in this integrant are shown in Figure 2D. The insertion point of human chromosome 17 is the coiled-coil domain-containing 57 (CCDC57) coding region. It has been reported that CCDC57 is one of the genes targeted by the integration of human papillomavirus 16 (HPV 16) [31].

### 3.3. Translocation of Chromosomes (5; 13) with HBV Integrants

**Figure 3:**
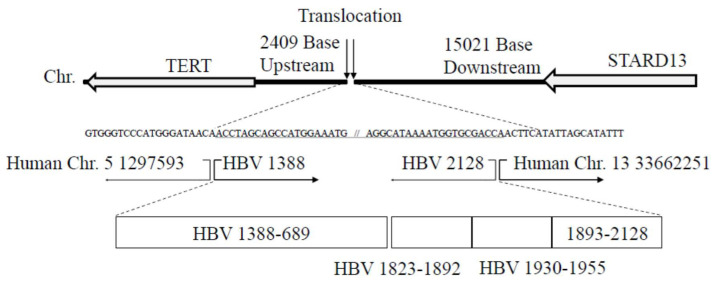
Translocation of chromosomes (5; 13) with hepatitis B virus (HBV) integrants. The results were confirmed by Sanger sequencing methods. Chr, chromosome (Hg19); TERT, telomerase reverse transcriptase; STARD13, StAR-related lipid transfer domain containing 13 genes.

We also observed the translocation of chromosomes (5; 13) with HBV genome integrants (Figure 3). The results confirmed by Sanger sequencing methods demonstrate that the insertion point in human chromosome 5 is 2409 bp upstream of the telomerase reverse transcriptase (TERT) gene, which is located near the TERT promoter region. The HBV regions included in this integrant are shown in Figure 3. The insertion point in human chromosome 13 is also 15021 bp downstream of the StAR-related lipid transfer domain containing 13 (STARD13) genes (Figure 3).

### 3.4. Automatic Expression of Proteins of HBV Integrants from Chromosomes 3 and 11, from the Vector without Any Promoter Sequences

**Figure 4:**
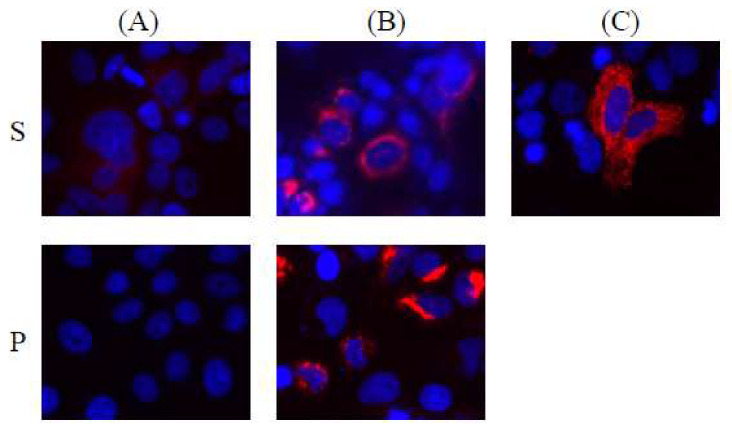
Automatic expression of proteins of HBV integrants from chromosomes 3 and 11. Representative images are shown (40×). Fluorescent immunostaining for HBsAg (**S**) and HBV polymerase (**P**) in PLC/PRF/5 cells (**A**), Huh7 cells transfected with the pCR-Blunt II-TOPO-HBV integrant from chromosome 3 (**B**), or Huh7 cells transfected with the pCR-Blunt II-TOPO-HBV integrant from chromosome 11 (**C**).

To examine the function of HBV integrant DNA from chromosome 3 in Huh7 cells after 24 h of transfection, we examined the expression of each HBV protein by immunofluorescence analysis. Compared to the expression of HBV proteins in PLC/PRF/5 (Figure 4A), we observed higher expression of both HBsAg and HBV polymerase protein in HBV integrant DNA-transfected Huh7 cells (Figure 4B). We did not observe HBcAg or HBx protein expression in HBV integrant DNA-transfected Huh7 cells (data not shown). 

After 24 h of transfection of the HBV integrant from chromosome 11 into Huh7 cells, we observed only HBsAg expression (Figure 4C). As the pCR-Blunt II-TOPO vector is a cloning vector and does not contain any promoters for the expression of coding proteins in mammalian cells, these integrants should automatically express the HBV-host fusion, HBsAg or HBV polymerase protein. No previous studies have shown polymerase expression in PLC/PRF/5 cells.

## 4. Discussion

We observed that HBV GT-A is a major GT of HBV integrated into PLC/PRF/5 cells. HBV sequence capture-based NGS is useful for the analysis of HBV genome integrants and their locations in the human genome. Among these HBV genome integrants, we performed functional analysis and demonstrated the automatic expression of some HBV proteins of HBV integrants from chromosomes 3 and 11 in Huh7 cells transfected with these DNA sequences.

The integration of a viral genome could lead to the disruption of the function of the human genome [32,33,34,35]. The deregulation of key cellular genes by HBV integration, which may present a selective growth advantage to hepatocytes and result in hepatocarcinogenesis, is thought to occur through several distinctive mechanisms.

PLC/PRF/5 cells can produce HBsAg in the cell culture medium [14,19]. Edman et al. reported that the PLC/PRF/5 cell line contains at least six (four complete and two incomplete) HBV genomes integrated into high-molecular-weight host DNA [20]. Northern blot analysis demonstrated the presence of RNA transcripts specific for the surface antigen sequences of HBV DNA and the absence of detectable transcripts corresponding to the hepatitis B core antigen, supporting the results of the immunofluorescence analysis conducted in the present study (Figure 4).

A previous in situ hybridization study with an HBV DNA probe for metaphase chromosomes of the PLC/PRF/5 cell line followed by statistical analysis identified 3 integration sites, namely, 11q22, 15q22-q23 and 18q12 [36], and we also found partial HBV genomes in chromosomes other than 11, 15, and 18 (Table 1). It is possible that our methods are more sensitive and less error-prone.

HBV genome integration into exons and introns results in truncated proteins and decreased protein expression levels, respectively. We found HBV integrants in the intron of chromosome 3 (Figure 2A) and part of the exon of CCDC57 in chromosome 17 (Figure 2C). We also found HBV integrants in the exon of SAC3D1 in chromosome 11 (Figure 2B).

Monjardino et al. reported that a defective HBV DNA molecule (approx. 2.8 kilobase pairs) appears to be integrated in a head-to-tail tandem arrangement, and they proposed that such defective molecules may be involved in the induction of hepatocarcinogenesis by HBV [26].

HBV genome integration induces aberrant promoter function in the host genome. Figure 3 shows HBV genome integration in a region close to the TERT promoter. Telomerase activity, which restores the length of telomere repeat arrays, is frequently observed in various malignancies, including HCC. As TERT is a protooncogene, the integration of HBV could result in hepatocarcinogenesis through its amplification and/or overexpression [37]. We also measured TERT mRNA by real-time RT-PCR, but we did not see any difference in TERT mRNA among PLC/PRF/5, Huh7, and HepG2 cells.

STARD13/Deleted in Liver Cancer (DLC) proteins belong to the RhoGAP family, and this protein is more abundantly expressed in HCC tissue, in particular, in the association with inflammation background [38]. STARD13 is related to HCC growth and hepatocarcinogenesis [38,39,40], although there is a report that HCC-patients with higher STARD13 or Fas expression levels have longer overall survival [41]. The consequences of the detected HBV integration sites and the clinical consequences or integration impact on hepatocarcinogenesis are yet unclear.

Interestingly, the automatic expression of some proteins encoded by HBV integrants from chromosomes 3 and 11 was observed in Huh7 cells transfected with these DNA sequences. This phenomenon may be involved in hepatocarcinogenesis in patients infected with HBV. Chromosome 11 HBV integrants did not code HBV full-length polymerase (Figure 2D). This may be the reason why we did not observe HBV polymerase protein in Huh7 cells transfected by chromosome 11 HBV integrants (Figure 4C). Further studies, including the functional analysis of these mechanisms, are needed.

In the present study, we enhanced NGS using HBV-targeted sequence capture. Although metagenomic shotgun sequencing is an important tool for the characterization of viral populations, metagenomic shotgun sequencing occasionally lacks sensitivity and may yield insufficient data for detailed analysis [42,43]. A targeted sequence-capture panel enhances metagenomic shotgun sequencing [42,43]. As genetic libraries must be generated from samples with low concentrations of HBV DNA and a high content of nucleic acids from a host in many cases [44], HBV hybridization-based enrichment may be useful for improving the sensitivity of the detection of HBV genome integration in hepatocytes. However, it is difficult to demonstrate how many copies of HBV deletion/rearrangements exist per cell, and single-cell genome sequencing may be helpful in this case [45].

Characteristics of human hepatoma PLC/PRF/5 cells have been reported for 40 years [36,46,47,48], and several genome sequences in the present study were not reported in detail. Watanabe et al. extensively analyzed the characteristics of human hepatoma PLC/PRF/5 cells using other NGS strategies [49]. However, they did not report the translocation of chromosomes (5; 13) with HBV integrants, which the present study has mentioned, suggesting that the HBV sequence capture-based NGS method is the more powerful and sensitive tool for the analysis of HBV integrants. 

Characteristics of human hepatoma PLC/PRF/5 cells may not reflect those of human HCC samples. It may be useful to analyze the HBV integrants in human HCC samples by our methods, although there may be false-positives in their analysis and their impact when real clinical samples that contain high levels of circulating virus are used. A similar approach has already been used for the identification of HBV integration in the human genome of clinical samples [12]. Compared to the method reported by Li et al. [12], our method does not require special computational analysis. The limitation of our study is that we did not use clinical samples. Further study is needed.

## 5. Conclusions

The HBV replication mechanism and the mechanism of integration, its functions, and the clinical impact on hepatocarcinogenesis remain largely unknown. HBV sequence capture-based NGS is a useful and powerful tool for the assessment of HBV genome integration to explore HBV genome integrants and their locations in the human genome. Clinical impact on hepatocarcinogenesis and new treatment strategies can be provided through this method.

## Figures and Tables

**Table 1:** The mapped reads and average coverage of hepatitis B virus (HBV) sequence capture-based next-generation sequencing in the present study.

Reference Sequence	Reference Length	Consensus Length	Total Reads	Single Reads	Reads in Pairs	Average Coverage
Chr. 1	2.49 × 10^8^	1,545,531	190,160	18,518	171,642	0.076008
Chr. 2	2.43 × 10^8^	1,344,151	171,652	23,708	147,944	0.06996
Chr. 3	1.98 × 10^8^	985,756	134,430	19,312	115,118	0.067181
Chr. 4	1.91 × 10^8^	763,084	101,024	27,548	73,476	0.051384
Chr. 5	1.81 × 10^8^	878,501	107,805	12,905	94,900	0.059238
Chr. 6	1.71 × 10^8^	978,370	143,424	13,186	130,238	0.083435
Chr. 7	1.59 × 10^8^	1,044,242	136,171	13,449	122,722	0.085094
Chr. 8	1.46 × 10^8^	762,738	93,417	14,827	78,590	0.063299
Chr. 9	1.41 × 10^8^	599,234	65,741	9779	55,962	0.046138
Chr. 10	1.36 × 10^8^	769,055	110,259	27,795	82,464	0.079924
Chr. 11	1.35 × 10^8^	863,578	2,006,583	126,939	1,879,644	1.488781
Chr. 12	1.34 × 10^8^	736,429	115,138	21,172	93,966	0.084807
Chr. 13	1.15 × 10^8^	412,243	58,815	10,367	48,448	0.050038
Chr. 14	1.07 × 10^8^	455,809	53,252	4762	48,490	0.049338
Chr. 15	1.03 × 10^8^	490,545	348,653	17,775	330,878	0.338605
Chr. 16	90,354,753	563,883	114,919	48,517	66,402	0.122922
Chr. 17	81,195,210	524,407	106,805	25,583	81,222	0.128115
Chr. 18	78,077,248	394,380	52,617	7057	45,560	0.067025
Chr. 19	59,128,983	447,625	53,263	7119	46144	0.089332
Chr. 20	63,025,520	474,048	68,184	5948	62,236	0.107619
Chr. 21	48,129,895	254,738	32,117	4913	27,204	0.0658
Chr. 22	51,304,566	253,101	29,371	3049	26,322	0.05685
Chr. X	1.55 × 108	674,067	73,717	8491	65,226	0.047146
Chr. Y	59,373,566	100,605	8838	8018	820	0.013505
Chr. MT	16,569	14,178	4540	172	4368	27.45869
HBV	3215	3213	1,784,734	448,408	1,336,326	53,732.01

Chr., chromosome (Hg19); MT, mitochondria; HBV, hepatitis B virus (AB014378) showing 90% (2915/3220) homology to full-length HBV sequence (PLC-HBV).

**Table 2:** HBV and human genomes in PLC/PRF/5 cells determined by HBV sequence capture-based next-generation sequencing.

Human Chromosome (Chr.)	Human Junction Nucleotide Position	Gene Name	HBV Fragment Start Position	HBV Fragment End Position	HBV Genome	Number of Total Reads *
Chr.3	131170552	NA	1044	1406	P, S, X	3673
Chr.3	131171957	NA	1415	1914	C, P, S, X	3589
Chr.4	181507570	NA	96	432	P, S	1548
Chr.4	181508764	NA	235	387	P, S	4377
Chr.5	1297593	NA	1175	1364	P, S	3680
Chr.8	35304663	UNC5D	2389	2862	C, P, S	4625
Chr.11	64808415	SNX15	1313	1575	P, S, X	5911
Chr.11	64808432	SAC3D1	2575	2851	P, S	6616
Chr.12	110012332	MVK	692	1379	P, S, X	10,130
Chr.13	33662251	NA	1897	2109	C, P, S	4060
Chr.13	33662698	NA	783	1612	P, S, X	1494
Chr.17	80063662	CCDC57	428	2586	P, S	7699
Chr.17	80065497	CCDC57	2062	2420	C, P, S	6918

Chr., chromosome (Hg19); NA, not available; UNC5D, unc-5 netrin receptor D; SNX15, sorting nexin 15; SAC3D1, SAC3 domain-containing 1; MVK, mevalonate kinase; CCDC57, coiled-coil domain-containing 57; HBV, hepatitis B virus; C, core; P, polymerase; S, surface antigen; X, HBx; *, confirmed by HBV sequence capture-based next-generation sequencing.
